# The A179L Gene of African Swine Fever Virus Suppresses Virus-Induced Apoptosis but Enhances Necroptosis

**DOI:** 10.3390/v13122490

**Published:** 2021-12-13

**Authors:** Jun Shi, Wei Liu, Miao Zhang, Jing Sun, Xiulong Xu

**Affiliations:** 1 Institute of Comparative Medicine, College of Veterinary Medicine, Yangzhou University, Yangzhou 225009, China; SJ1277383407@126.com (J.S.); liuw@yzu.edu.cn (W.L.); 18762314152@163.com (M.Z.); sunj@yzu.edu.cn (J.S.); 2 Jiangsu Co-Innovation Center for Prevention and Control of Important Animal Infectious Diseases and Zoonosis, Yangzhou University, Yangzhou 225009, China

**Keywords:** African swine fever virus, *A179L*, apoptosis, necroptosis

## Abstract

A179L, a non-structural protein of African swine fever virus (ASFV), is capable of suppressing apoptosis by binding the BH3 domain of the pro-apoptotic Bcl-2 family proteins via a conserved ligand binding groove. Our present study aims to determine if A179L affects necroptosis, the second form of programmed cell death induced by DNA and RNA viruses. Here we report that A179L enhanced TNF-α or TSZ (TNF-α, Smac, and Z-Vad)-induced receptor-interacting protein kinase (RIPK1), RIPK3, and mixed lineage kinase domain like peudokinase (MLKL) phosphorylation in L929 cells, a murine fibrosarcoma cell line. Sytox green staining revealed that A179L significantly increased the number of necroptotic cells in TSZ-treated L929 cells. Using human herpes simplex virus 1 (HSV-1) to model DNA virus-induced cell death, we found that A179L blocked the HSV-1-induced cleavage of poly (ADP-ribose) polymerase (PARP), caspase 8, and caspase 3 and decreased the number of apoptotic cells in HSV-1-infected IPEC-DQ cells, a porcine intestinal epithelial cell line. In contrast, A179L transfection of IPEC-DQ cells enhanced HSV-1-induced RIPK1, RIPK3, and MLKL phosphorylation and increased the number of necroptotic cells. Consistently, A179L also suppressed apoptosis but enhanced the necroptosis induced by two RNA viruses, Sendai virus (SeV) and influenza virus (IAV). Our study uncovers a previously unrecognized role of A179L in regulating cell death and suggests that A179L re-directs its anti-apoptotic activity to necroptosis.

## 1. Introduction

African swine fever (ASF) is an acute and highly contagious infectious disease caused by the ASF virus (ASFV) [1]. ASF outbreak was first reported in August 2018 in Shenyang, Liaoning Province in China and then spread rapidly to many other regions, causing a devastating impact on the pig industry [2]. ASFV is a double-stranded DNA virus that is the sole characterized member of the *asfarviridae* family [3]. Its genome is 170–193 kb long and it harbors 150–167 open reading frames (ORFs) [3]. These ORFs encode >50 structural and >100 nonstructural proteins. Many nonstructural proteins interfere with the immune response and perturb cell death [4,5]. Several recent studies have shown that ASFV nonstructural proteins such as MGF-505-7A, pMGF505-7R, A276R, I226R, I177L, and A528R can block IRF3 activation and suppress the antiviral innate immunity [5,6,7,8,9,10,11,12]. The functions of many remaining nonstructural genes of ASFV remain to be characterized [13]. 

Apoptosis is considered the first-line defense of host cells to restrict virus replication [14]. Both RNA and DNA viruses can induce apoptosis [14]. However, the mechanisms of virus-induced cell death are complex and incompletely understood [15]. Sendai virus activates retinoic acid-inducible gene I (RIG-I), leading to its conformation change and binding to mitochondrial antiviral signaling protein (MAVS), which then recruits and activates caspase 8 through the caspase recruitment domain (CARD) domains to initiate apoptosis [15]. Influenza virus-induced apoptosis involves viral RNA-activated Z-DNA binding protein 1 (ZBP1) to form a complex with RIPK3 and caspase 8 [16,17]. In addition, both DNA and RNA viruses may induce apoptosis by up-regulating Fas expression and by their viral proteins. For example, the PB1-F2 protein of IAV can target the mitochondrial proteins, adenine nucleotide translocase 3 (ANT3) and voltage-dependent anion channel 1 (VDAC1), to release cytochrome C and induce intrinsic apoptosis [18,19]. HSV-1 induces apoptosis through its ICP0 protein [20]. In addition, activation of IRF3 in virus-infected cells may also induce apoptosis by facilitating the insertion of Bax into the mitochondria. However, many viruses, such as murine cytomegalovirus, vaccina, and cowpox viruses express a caspase inhibitor to subvert apoptosis [14,21,22,23]. As a large DNA virus, several nonstructural proteins of ASVF, such as A179L, A224L, DP71L, and EP153R, are capable of inhibiting apoptosis at the early stage of virus infection and facilitate virus replication [24]. The p54 structural protein can also promote apoptosis in the later stage of virus infection [24,25]. A179L, a nonstructural protein of ASFV, is capable of blocking apoptosis induced by truncated Bid (tBid) or baculovirus [24]. A179L interacts with the BH3-containing proteins, such as tBid, Bax, and Bak, inhibiting tBid-induced apoptosis in Vero cells and baculovirus-induced apoptosis in Sf9 cells, an insect cell line [26,27,28,29]. However, whether A179L affects apoptosis induced by ASFV or other DNA viruses remains unexplored. In addition to its role in apoptosis, A179L interacts with Beclin 1 through its BH3 homology domain and inhibits starvation-induced autophagosome formation [30,31]. Interestingly, Shimmon recently reported that A179L is dispensable in ASFV-mediated suppression of starvation-induced autophagy in Vero cells [32].

Necroptosis is another important form of programmed cell death. When apoptosis is inhibited, necroptosis becomes the second-line defense against intracellular pathogens [33]. In addition, necroptosis causes a significant inflammatory response, which in turn alerts the immune system to assist virus clearance [33]. Necroptosis induced by the TNF-α receptor relies primarily on the formation of a cytosolic complex consisting of TNF receptor-associated factor (TRAF), TNFR1 associated death domain protein (TRADD), caspase 8, and RIPK1 [33]. RIPK1 activation leads to the phosphorylation and activation of RIPK3, which then phosphorylates MLKL and induces its oligomerization translocation from the cytoplasm to the cell membrane [33]. The MLKL oligomer is an ion channel that allows calcium ions to enter cells to increase intracellular osmotic pressure, leading to cell membrane disruption and ultimately cell necroptosis [33]. DNA and RNA viruses induce necroptosis by different mechanisms. For example, ICP6, an early gene product of HSV-1, is an RIP homotypic interaction motif (RHIM)-containing protein that binds to murine RIPK3 and induces necroptosis. The viral RNA of IAV binds ZBP1 to activate RIPK3 and induce necroptosis [16,17].

It is well established that inhibition of caspase 8 activity switches TNF-α-induced apoptosis to necroptosis. Though A179L inhibits apoptosis in multiple settings, whether A179L can inhibit virus-induced caspase activation to enhance necroptosis remains unexplored. Here we report that A179L suppresses apoptosis induced by a DNA virus (HSV-1) and two RNA viruses (IAV and Sendai virus). In contrast, A179L enhanced necroptosis induced by TSZ and by these three types of viruses. Our study reveals a novel function of the *A179L* gene and suggests that host cells may use an alternative form of programmed cell death to attenuate virus replication. 

## 2. Materials and Methods

### 2.1. Reagents 

SMAC (AT-406) (#HY-15454) and z-VAD(OMe)-FMK (#HY-16658) were purchased from MedChemExpress LLC (Shanghai, China). Sytox green (SYTOX^®^ Green Nucleic Acid Stain) (#S7020) was purchased from Invitrogen Inc. (Carlsbad, CA, USA). FITC Annexin V Apoptosis Detection Kit (Cat# 556547) was purchased from BD Pharmingen (Shanghai, China). Antibodies against human phospho-RIPK1^S166^ (#65746), murine phospho-RIPK1^S166^ (#31122), RIPK1 (#3493), human phospho-RIPK3^S227^ (#93654), murine RIPK3 (#95702), human RIPK3 (#13526), murine MLKL (#37705), human MLKL (#14993), caspase-3 (#14220), cleaved caspase-3 (#9664), caspase-8 (#4790), cleaved human caspase-8 (#9496S), cleaved mouse caspase-8 (#8592), PARP (#9532S), and anti-FLAG antibody (#8146) were purchased from Cell Signaling Technology, Inc. (Danvers, MA, USA). Antibodies against β-actin (#sc-47778) and glyceraldehyde 3-phosphate dehydrogenase (GAPDH) were purchased from Santa Cruz Biotechnology Inc. (San Diego, CA, USA). Antibodies against murine phospho-MLKL (#ab196436), human phospho-MLKL^S358^ (#ab187091), and murine phospho-RIPK3^T231/S232^ (#ab222320) were purchased from Abcam Inc. (Shanghai, China). The anti-GFP (Code#HT801 Lot#O21209) mouse monoclonal antibody was purchased from TransGen Biotech (Beijing, China). Recombinant murine TNF-α (#410-MT-010) was purchased from R and D Systems, Inc. (Shanghai, China).

### 2.2. Plasmid DNA

The nonstructural genes of ASFV were amplified by using the homogenized sample of ASFV-infected spleen tissue as the template. The primers used in the PCR reaction for amplifying A179L were 5′-CCCAAGCTGGCTAGCGCAATGGAGGGAGAA-GAGTTAATATATC-3′ (*Nhe* I) and 5′-TTTGTAGTCGGATCCTATCAAATTGCAGTTTCTTAATAACTG-3 (*Bam* HI). The primers for amplifying DP71L were 5′-CCCAAGCTGGCTAGCGCAATGGGGAGGCGGCGCAAAAAAC-3′ (*Nhe* I) and 5′-TTTGTAGTCGGATCCCTGCTGCTCCAGTAGCTT -3 (*Bam* HI). The primers used for amplifying A238L were 5′-CCCAAGCTGGCTAGCGCAATGTTTCCAGAAAGGGAGATAG-3′ and 5′-TTTGTAGTCGGATCCCACACAGGAACTTTGGAATTCTG-3′. The PCR fragments were purified and cloned into the Nhe I- and Bam HI-cleaved pcDNA3.1-FLAG vector by homologous recombination. The expression vectors encoding these three ASFV genes were validated by sequencing. 

### 2.3. Cell Lines and Viruses

L929 (a murine fibrosarcoma cell line), NL20 (a human non-tumoral bronchial epithelial cell line), and Vero (CCL-81) cells were purchased from the American Tissue Culture Collection (Manassas, VA). The L929 cells were grown in DMEM containing 10% fetal bovine serum (FBS). The NL20 cells were grown in F12 containing 4% fetal bovine serum (FBS). The Vero cells were grown in complete αMEM medium containing 10% fetal bovine serum, streptomycin, and penicillin (100 U/mL), sodium pyruvate (1 mM), and L-glutamine (2 mM). The IPEC-DQ cells, a subclone of the porcine intestinal epithelial cell line IPEC-J2, were kindly provided by Dongwan Yoo, College of Veterinary Medicine, the University of Illinois at Urbana-Champaign, Urbana, IL, USA. The HSV-1 (KOS strain) and A/PR8/34 (H1N1) virus were kindly provided by Liqian Zhu (College of Veterinary Medicine, Yangzhou University, Yangzhou, Jiangsu province, China). The GFP-tagged HSV-1 (GFP is fused with the VP26 protein of the KOS strain) was kindly provided by Zengfan Jiang (Peking University, Beijing, China). The Sendai virus was kindly provided by Chan Ding (Shanghai Veterinary Research Institute, Shanghai, China). The GPF-tagged Sendai virus was kindly provided by Feng Ma (Suzhou Institute of Systems Medicine, Suzhou, Jiangsu province, China). The HSV-1 was propagated and expanded in the Vero cells. The H1N1 and Sendai viruses were prepared by inoculating 10-day-old specific-pathogen-free embryonic chicken eggs. The Sendai virus and IAV titers were determined by a 10-fold serial dilution (10^1^ to 10^9^, and each dilution (10^5^–10^9^) in Vero and MDCK cells, respectively. The 50% tissue culture infection dose (TCID_50_/100 μL) values were determined by using the standard Reed and Muench method.

### 2.4. Western Blot

Cells grown in 6-well plates were harvested and lysed in NP-40 lysis buffer (50 mM Tris-HCl (pH 8.0), 150 mM NaCl, 1% NP-40, 5 mM EDTA, 10 µg/mL aprotinin, 10 µg/mL leupeptin, and 1 mM phenylmethylsulphonyl fluoride). After incubation on ice for 30 min, the cell lysates were prepared by spinning down at 4 °C, 15,000 rpm for 15 min. The cell lysates were analyzed by Western blot with antibodies against the proteins of interest, followed by horseradish peroxidase-conjugated goat anti-rabbit or anti-mouse IgG and SuperSignal Western Pico enhanced chemiluminescence substrate (Pierce Chemical Co., Rockford, IL, USA). The density of the bands was analyzed using NIH Image-J software (NIH, Bethesda, MD, USA) (https://imagej.nih.gov/ij/, accessed on 27 August 2021) and normalized by the arbitrary units of their corresponding total proteins or β-actin, as indicated. Quantified results were presented as the mean ± standard deviation (SD) from three experiments in the bar graphs. The original Western blot images can be found in Supplementary Materials File S1.

### 2.5. Flow Cytometry

The L929 cells seeded in 6-well plates were infected with HSV-1 or Sendai virus (1 MOI each). After incubation for 24 h, single-cell suspensions were prepared and stained for necroptosis by Sytox green or apoptosis with propidium iodide (PI) and Annexin V by using an FITC Annexin V Apoptosis Detection kit following the manufacturer’s instructions. The NL20 cells infected with IAV were similarly analyzed for apoptosis and necroptosis. Single-cell suspensions were run in a Beckman Coulter flow cytometer (Model CyAn ADP). The fluorescence intensity was analyzed using FlowJo software. The Sytox green- or Annexin-positive cells were gated. The percentage of Sytox green- or Annexin V-positive cells from three independent experiments were calculated and statistically analyzed using the unpaired Student’s *t* test. The data in bar graphs are the mean ± SD of three independent experiments.

### 2.6. Statistical Analysis 

The differences among the Western blot band densities and the percent of apoptotic or necroptotic cells were statistically analyzed using an unpaired Student *t* test. A *p* value of <0.05 was considered statistically significant. All statistical analyses were performed with GraphPad Prism (GraphPad software 8.0 (https://www.graphpad.com/scientific-software/prism, accessed on 27 August 2021).

## 3. Results 

### 3.1. A179L Enhances TSZ-Induced Necroptosis

**Figure 1:**
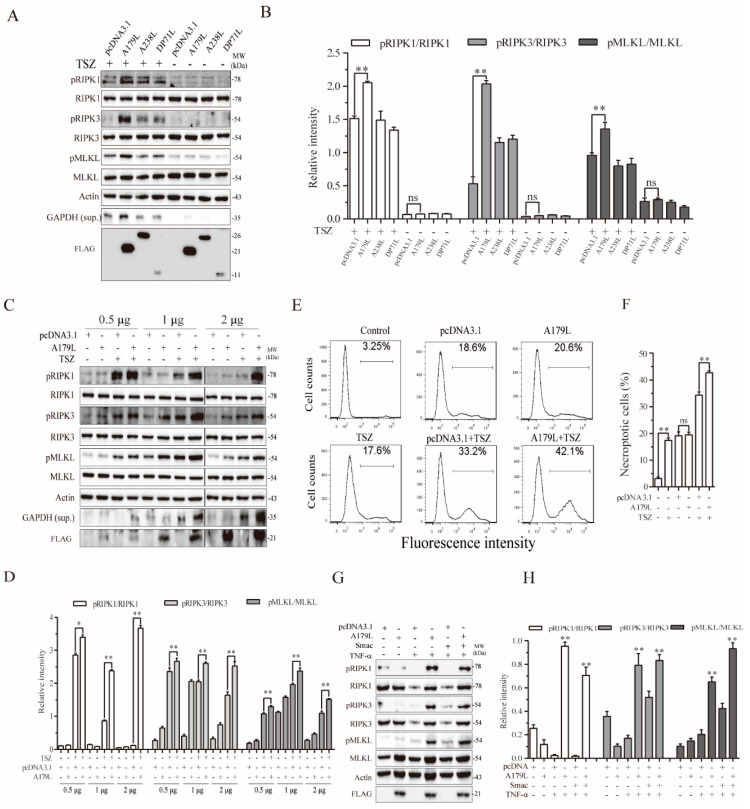
A179L enhances TSZ-induced necroptosis. L929 cells seeded in a 12-well plate were transiently transfected with pcDNA3.1 or the vector encoding the three indicated nonstructural genes of ASFV (2 μg/each) (**A**). Alternatively, L929 cells seeded in a 12-well plate were transiently transfected with pcDNA3.1 (2 μg) or the indicated amount of the A179L expression vector (**C**). After incubation for 36 h, the cells were left untreated or treated with TSZ (TNF-α, 20 ng/mL; Smac,100 nM; and Z-VAD,30 μM) for 2 h. Cell lysates were prepared and analyzed for the levels of RIPK1^S166^, RIPK3, MLKL, and their corresponding total proteins. The levels of the ASFV genes were probed with an anti-FLAG antibody. GAPDH in the conditioned media of L929 cells was also probed. The density of the bands was analyzed by using NIH Image-J software and normalized by the arbitrary units of their corresponding total protein or β-actin (**B**,**D**). Data are the mean ± SD of three experiments. * p < 0.05, ** p < 0.01, compared to the pcDNA3.1 control; ns, not significant. (**E**) L929 cells seeded in a 6-well plate were left untransfected or were transiently transfected with pcDNA3.1 or the indicated amount of the A179L expression vector (4 μg each). After incubation for 36 h, the cells were left untreated or treated with TSZ for 2 h. Single-cell suspensions were stained for necroptosis with Sytox green (**E**). The results represent the mean percent of cell death ± SD of three independent experiments (**F**). * *p* < 0.05, ** *p* < 0.01. (**G**) L929 cells seeded in a 12-well plate were transiently transfected with pcDNA3.1 or the A179L expression vector (2 μg each). After incubation for 36 h, the cells were left untreated or were treated with TNF-α (20 ng/mL) or Smac (100 nM) for 2 h. Cell lysates were prepared and analyzed for the phosphorylation of necroptosis-related protein as above. Relative protein phosphorylation was quantified by scanning the blots with NIH Image-J software and data were normalized by the arbitrary units of their corresponding total protein. (**H**) Data are the mean ± SD of three experiments. * p < 0.05, ** p < 0.01, compared to the pcDNA3.1 control.

We first investigated the effect of A179L, DP71L, and A238L, three nonstructural proteins of ASFV, on TSZ-induced necroptosis in L929 cells. DP71L recruits protein phosphatase 1 (PP1) to dephosphorylate elF2a and inhibit the activation of proapoptotic CHOP [34,35]. A238L interacts with the histone acetyltransferase p300/CBP and inhibits NF-κB activation [6]. L929 cells were chosen for our experiments because they readily undergo necroptosis by various stimuli and have been widely used to studying the mechanism of TNF-α-induced necroptosis [36,37]. L929 cells were mock-transfected or transfected with pcDNA3.1 or the vector encoding *DP71L*, *A238L*, and *A179L*, and then stimulated with TSZ for 2 h. As shown in Figure 1A,B, RIPK1, RIPK3, and MLKL phosphorylation was significantly enhanced in the TSZ-treated L929 cells by A179L, but not by A238L and DP71L. These three genes did not affect the basal levels of RIPK1, RIPK3, and MLKL phosphorylation in untreated cells. Necroptotic L929 cells release glyceraldehyde 3-phosphate dehydrogenase (GAPDH) into their conditioned media [38]. GAPDH in the conditioned media of L929 cells can serve as a marker of necroptosis [38]. As shown in Figure 1A, TSZ increased the levels of GAPDH expression released in the conditioned media of L929 cells. A179L significantly increased the levels of GAPDH expression in the conditioned media of TSZ-treated L929 cells, compared to those treated with TSZ alone. A179L, A238L, and DP71L expressions were confirmed by Western blot, showing molecular masses of 25, 20, and 15 kDa, respectively (Figure 1A). We next confirmed the specificity of A179L to enhance TSZ-induced necroptosis. As shown in Figure 1C,D, the levels of RIPK1, RIPK3, and MLKL phosphorylation in untreated L929 cells transfected with various amounts of A179L plasmid DNA were slightly higher than those transfected with the pcDNA3.1 vector. However, the levels of RIPK1, RIPK3, and MLKL phosphorylation in A179L-transfected L929 cells treated with TSZ were significantly higher than their corresponding pcDNA3.1-transfected controls (Figure 1C,D). The magnitude of A179L to increase RIPK1, RIPK3, and MLKL phosphorylation was dose-dependent (Figure 1C,D). 

We next conducted a flow cytometric analysis of Sytox green-stained cells to verify the stimulatory effect of A179L on TSZ-induced necroptosis. As shown in Figure 1E, the basal number of Sytox green-positive cells in the control was relatively low, only approximately 3% of cells spontaneously underwent necroptosis (Figure 1D). pcDNA3.1 and A179L transfection alone moderately increased the number of necroptotic cells, resulting in approximately 18–20% Sytox green-positive cells, which was almost equivalent to the untransfected cells treated with TSZ. The number of necroptotic cells in the A179L-transfected L929 cells treated with TSZ was significantly higher than those in the pcDNA3.1-transfected cells treated with TSZ (Figure 1D,E). These observations collectively suggest that A179L specifically enhances TSZ-induced necroptosis. 

We further investigated if A179L was able to sensitize L929 cells to TNF-α-induced necroptosis. L929 cells transfected with pcDNA3.1 vector or A179L were treated with TNF-α and/or Smac. As shown in Figure 1G, TNF-α alone or in combination with Smac induced little RIPK1, RIPK3, and MLKL phosphorylation in pcDNA3.1-transfected cells, but dramatically induced phosphorylation of these proteins in A179L-transfected cells. A179L did not further increase RIPK1, RIPK3, and MLKL phosphorylation in L929 cells treated with TNF-α plus Smac, compared to that treated with TNF-α alone. These observations suggest that the stimulatory effect of A179L on TNF-α-induced necroptosis is physiologically relevant.

### 3.2. A179L Suppresses HSV-1-Induced Apoptosis 

**Figure 2:**
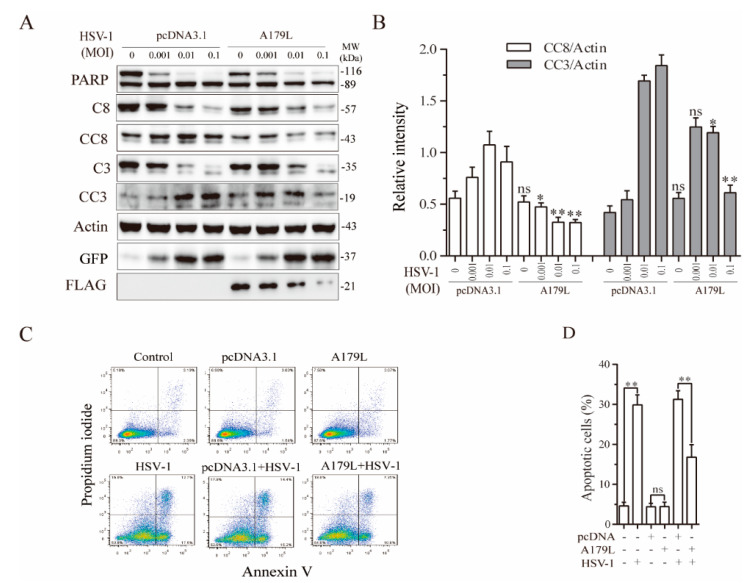
A179L suppresses HSV-1-induced apoptosis. (**A**) IPEC-DQ cells seeded in a 12-well plate were transiently transfected with pcDNA3.1 or the A179L expression vector. After incubation for 24 h, the cells were left uninfected or infected with HSV-1 (1 MOI) and then incubated for 24 h. Cell lysates were prepared and analyzed for the levels of PARP, caspase 3, and caspase 8 cleavage. A179L expression was monitored with an anti-FLAG antibody. The levels of HSV-1 infection were monitored with an anti-EGFP antibody. The densities of the bands were analyzed by using NIH Image-J software and normalized by the arbitrary units of their corresponding total protein or β-actin (**B**). Data are the mean ± SD of three experiments. * p < 0.05, ** p < 0.01, compared to the pcDNA3.1 control; ns, not significant. (**C**) IPEC-DQ cells seeded in a 6-well plate were left untransfected or transiently transfected with pcDNA3.1 or the A179L expression vector (2 μg each). After incubation for 24 h, the cells were left uninfected or infected with HSV-1 (1 MOI) for 24 h. Single-cell suspensions were stained with propidium iodide and Annexin V. Apoptosis was analyzed by flow cytometry. The results represent the mean percent of cell death ± SD of three independent experiments (**D**). * *p* < 0.05, ** *p* < 0.01.

It has been well established that many DNA viruses can induce both apoptosis and necroptosis [15,39]. The ICP6 gene of HSV-1 inhibits apoptosis and necroptosis in human cells but induces necroptosis in murine cells [20]. Since ASFV is a double-stranded DNA virus, we used HSV-1 as a model to determine if A179L also affected virus-induced cell death in IPEC-DQ cells, a porcine intestinal epithelial cell line. The main reason that we switched L929 cells to IPEC-DQ cells is that L929 cells, as a murine fibrosarcoma cell line produce interferons after transfection, which significantly inhibit virus replication. In addition, ASFV is a pathogen that primarily infects cells of swine origin. We first investigated the effect of A179L on HSV-1-induced apoptosis. As shown in Figure 2A, GFP-tagged HSV-1 infection led to the cleavage of PARP, caspase-8, and caspase-3 in a dose-dependent manner in pcDNA3.1-transfected IPEC-DQ cells. However, the magnitude of caspase-8 and caspase-3 cleavage was significantly lower in A179L-transfected cells than in pcDNA3.1-transfected cells (Figure 2B). The magnitude of PARP cleavage in A179L-transfected cells was also slightly lower than the pcDNA3.1transfected cells. Flow cytometric analysis revealed that pcDNA3.1 or A179L transfection alone did not significantly increase the number of apoptotic cells (Figure 2C). HSV-1 infection alone dramatically increased the number of apoptotic cells (Figure 2C). A179L transfection decreased the number of apoptotic cells in HSV-1-infected cells, compared to those transfected with the pcDNA3.1 vector (Figure 2C,D). 

### 3.3. A179L Enhances HSV-1-Induced Necroptosis

**Figure 3:**
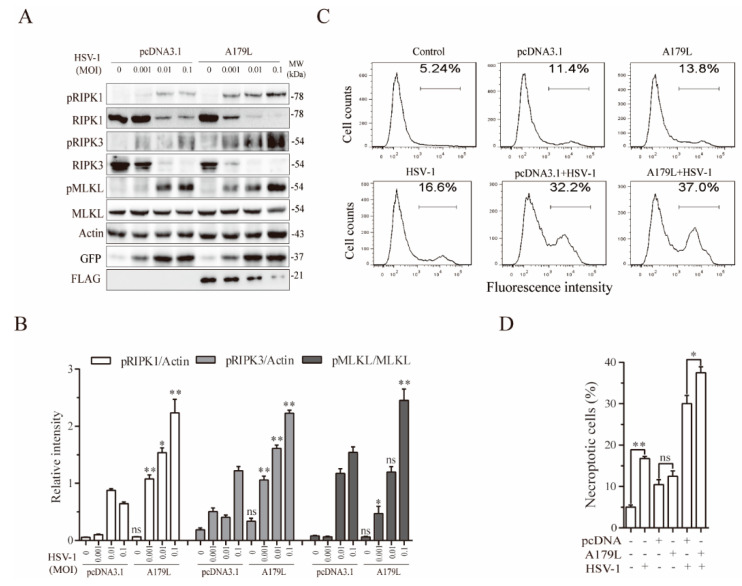
A179L enhanced HSV-1-induced necroptosis. (**A**) IPEC-DQ cells seeded in a 12-well plate were transiently transfected with pcDNA3.1 or the A179L expression vector. After incubation for 24 h, the cells were left uninfected or infected with the indicated dose of HSV-1 and then incubated for 24 h. Cell lysates were prepared and analyzed for the levels of RIPK1^S166^, RIPK3, and MLKL and their corresponding total proteins. A179L expression was probed with an anti-FLAG antibody. HSV-1 infection was monitored by Western blot with an anti-EGFP antibody. The density of the bands was analyzed by using NIH Image-J software and normalized by the arbitrary units of their corresponding total protein or β-actin. Data are the mean ± SD of three experiments (**B**). * p < 0.05, ** p < 0.01, compared to the pcDNA3.1 control; ns, not significant. (**C**) IPEC-DQ cells seeded in a 6-well plate were left untransfected or transiently transfected with pcDNA3.1or the indicated amount of the A179L expression vector (4 μg each). After incubation for 24 h, the cells were left uninfected or infected with HSV-1 (1 MOI) for 24 h. Single-cell suspensions were stained with Sytox green and analyzed for necroptosis by flow cytometry (**C**). The results represent the mean percent of cell death ± SD of three independent experiments (**D**). * *p* < 0.05, ** *p* < 0.01.

HSV-1-induced necroptosis in murine cells is independent of ZBP1 [20]. Instead, ICP6 binds to and activates RIPK3 to induce necroptosis [20]. We next determined if A179L enhanced HSV-1-induced necroptosis in IPEC-DQ cells. As shown in Figure 3A, HSV-1 infection dose-dependently increased the levels of RIPK1, RIPK3, and MLKL phosphorylation in pcDNA3.1-transfected L929 cells. The magnitude of RIPK1, RIPK3, and MLKL phosphorylation induced by HSV-1 infection was significantly higher in A179L-transfected cells than in pcDNA3.1-transfected controls (Figure 3B). Interestingly, the levels of total RIPK1 and RIPK3 proteins were dose-dependently decreased by HSV-1 infection in both pcDNA3.1- and A179L-transfected cells (Figure 3A). The magnitude of RIPK1 and RIPK3 cleavage was slightly higher in the A179L-transfected IPEC-DQ cells than in pcDNA3.1-transfected controls. HSV-1 infection also dose-dependently decreased the levels of A179L protein (Figure 3A). Flow cytometric analysis of Sytox green-stained cells revealed that pcDNA3.1 and A179L transfection alone slightly but significantly increased the number of necroptotic IPEC-DQ cells (Figure 3C,D). HSV-1 infection alone remarkably increased the number of Sytox green-positive cells. A179L transfection further increased the number of necroptotic cells following HSV-1 infection, compared to its corresponding pcDNA3.1 control (Figure 3C,D).

### 3.4. A179L Suppresses Sendai Virus-Induced Apoptosis

**Figure 4:**
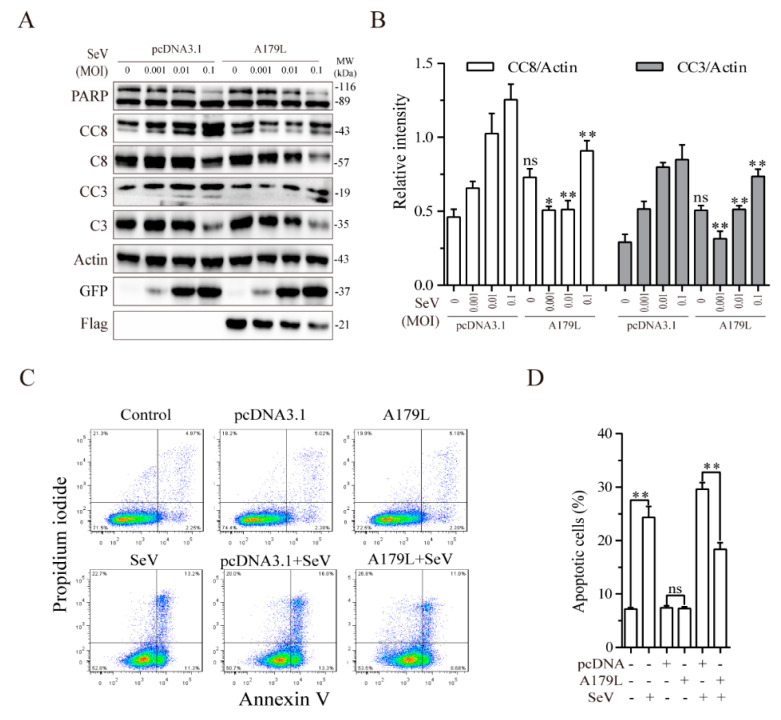
A179L suppresses Sendai virus-induced apoptosis. (**A**) IPEC-DQ cells seeded in a 12-well plate were transiently transfected with pcDNA3.1 or the A179L expression vector. After incubation for 24 h, the cells were left uninfected or infected with Sendai virus and then incubated for 24 h. Cell lysates were prepared and analyzed for levels of PARP, caspase 3, and caspase 8 cleavage. A179L expression was probed with an anti-FLAG antibody. Sendai virus infection was monitored by Western blot with an anti-EGFP antibody. The density of the bands was analyzed by using NIH Image-J software and normalized by the arbitrary units of their corresponding total protein or β-actin. Data are the mean ± SD of three experiments (**B**). * p < 0.05, ** p < 0.01, compared to the pcDNA3.1 control; ns, not significant. (**C**) IPEC-DQ cells seeded in a 6-well plate were left untransfected or transiently transfected with pcDNA3.1or the indicated amount of the A179L expression vector (4 μg each). After incubation for 24 h, the cells were left uninfected or infected with HSV-1 (1 MOI) for 24 h. Single-cell suspensions were stained with propidium iodide and Annexin V. Apoptosis was analyzed by flow cytometry (**C**). The results represent the mean percent of cell death ± SD of three independent experiments (**D**). * *p* < 0.05, ** *p* <0.01.

Having shown that A179L suppressed apoptosis but facilitated necroptosis induced by HSV-1, we next determined if A179L had similar effects on RNA virus-induced cell death. Sendai virus, a single-stranded RNA virus belonging to the family of *paramyxoviridae*, readily induces both apoptosis and necroptosis [23]. We first investigated the effect of A179L on Sendai virus-induced apoptosis. As shown in Figure 4A, Sendai virus infection led to the cleavage of caspase-8 and caspase-3 in a dose-dependent manner in pcDNA3.1-transfected IPEC-DQ cells. The magnitude of the caspase-8 and caspase-3 cleavage induced by Sendai virus infection was significantly lower in A179L-transfected cells than in pcDNA3.1-transfected cells (Figure 4B). Flow cytometric analysis revealed that pcDNA3.1 or A179L transfection alone did not significantly affect apoptosis, as the number of Annexin V-positive cells was almost equivalent to those of the untransfected control (Figure 4C,D). A179L transfection significantly decreased the number of apoptotic cells infected with Sendai virus, compared to those transfected with the pcDNA3.1 vector control (Figure 4C,D).

### 3.5. A179L Enhances Sendai Virus-Induced Necroptosis

**Figure 5:**
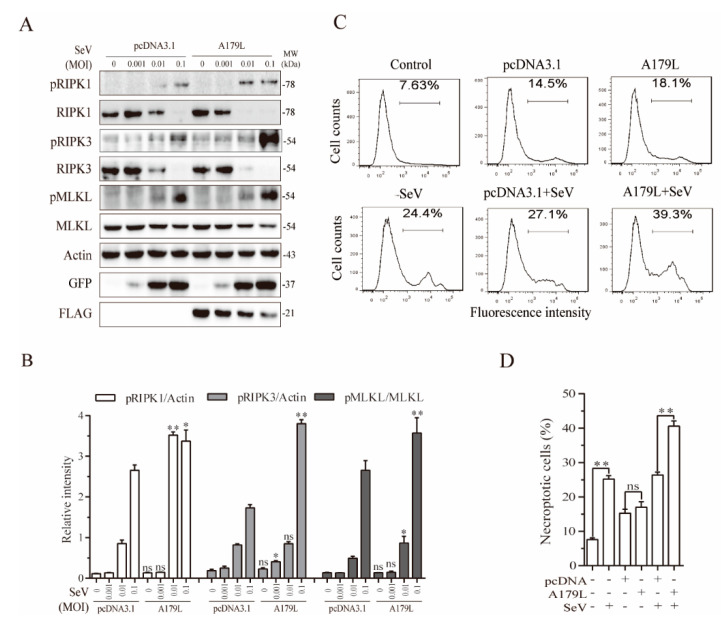
A179L enhanced Sendai virus-induced necroptosis. (**A**) IPEC-DQ cells seeded in a 12-well plate were transiently transfected with pcDNA3.1 or the A179L expression vector. After incubation for 24 h, the cells were left uninfected or infected with the indicated dose of Sendai virus and then incubated for 24 h. Cell lysates were prepared and analyzed for the levels of RIPK1^S166^, RIPK3, MLKL, and their corresponding total proteins. A179L expression was analyzed by Western blot with an anti-FLAG antibody. Sendai virus infection was monitored by Western blot with an anti-EGF antibody. The densities of the bands were analyzed by using NIH Image-J software and normalized by the arbitrary units of their corresponding total protein or β-actin. Data are the mean ± SD of three experiments (**B**). * p < 0.05, ** p < 0.01, compared to the pcDNA3.1 control; ns, not significant. (**C**) IPEC-DQ cells seeded in a 6-well plate were left untransfected or transiently transfected with pcDNA3.1or the indicated amount of the A179L expression vector (4 μg each). After incubation for 24 h, the cells were left uninfected or infected with Sendai virus (1 MOI) for 24 h. Single-cell suspensions were stained with Sytox green and analyzed for necroptosis by flow cytometry (**C**). The results represent the mean percent of cell death ± SD of three independent experiments (**D**). * *p* < 0.05, ** *p* < 0.01.

We next determined if A179L also enhanced Sendai virus-induced necroptosis. As shown in Figure 5A, Sendai virus infection dose-dependently increased the levels of RIPK1 and MLKL phosphorylation in pcDNA3.1-transfected IPEC-DQ cells. The magnitude of RIPK1 and MLKL phosphorylation was significantly higher in the A179L-transfected cells than in the pcDNA3.1-transfected controls infected with Sendai virus (Figure 5B). Interestingly, the levels of the RIPK1 and RIPK3 proteins were dose-dependently decreased by Sendai virus infection in both the pcDNA3.1- and A179L-transfected cells. The magnitude of RIPK1 and RIPK3 degradation was slightly higher in the A179L-transfected IPEC-DQ cells than in the pcDNA3.1-transfected controls. Sendai virus infection also dose-dependently decreased the levels of A179L protein. Flow cytometric analysis of Sytox green-stained cells revealed that pcDNA3.1 and A179L transfection alone slightly increased the number of necroptotic IPEC-DQ cells (Figure 5C,D). HSV-1 infection alone significantly increased the number of Sytox green-positive cells. A179L transfection further increased the number of necroptotic cells following Sendai virus infection, compared to the pcDNA3.1 vector control (Figure 5C,D).

### 3.6. A179L Suppresses Influenza Virus-Induced Apoptosis

**Figure 6:**
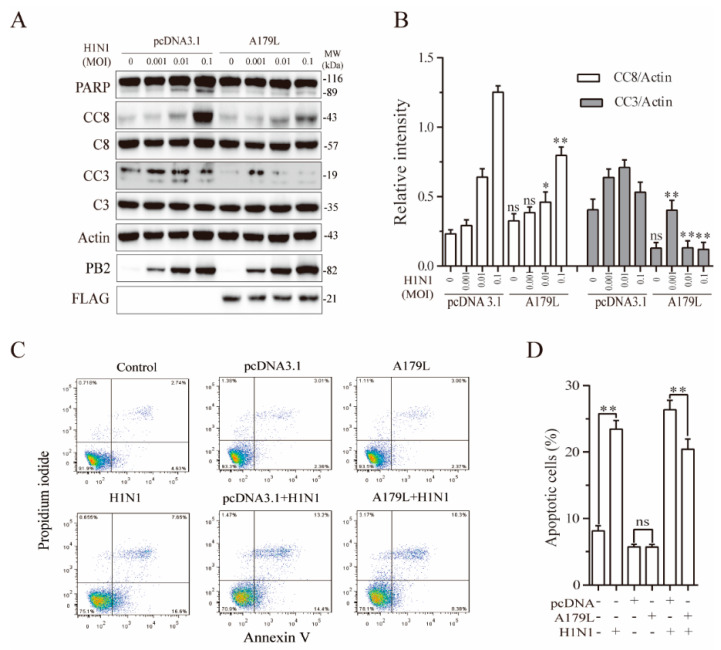
A179L suppresses IAV-induced apoptosis. (**A**) NL20 cells seeded in a 12-well plate were transiently transfected with pcDNA3.1 or the A179L expression vector. After incubation for 24 h, the cells were left uninfected or infected with IAV and then incubated for 24 h. Cell lysates were prepared and analyzed for their levels of PARP, caspase 3, and caspase 8 cleavage. A179L expression was probed with an anti-FLAG antibody. IAV infection was monitored by Western blot with an anti-PB2 antibody. The density of the bands was analyzed using NIH Image-J software and normalized by the arbitrary units of their corresponding total protein or β-actin. Data are the mean ± SD of three experiments (**B**). * p < 0.05, ** p < 0.01, compared to the pcDNA3.1 control; ns, not significant. (**C**) NL20 cells seeded in a 6-well plate were left untransfected or transiently transfected with pcDNA3.1or the indicated amount of the A179L expression vector (4 μg each). After incubation for 24 h, the cells were left uninfected or infected with IAV (1 MOI) for 24 h. Single-cell suspensions were stained with propidium iodide and Annexin V. Apoptosis was analyzed by flow cytometry (**C**). The results represent the mean percent of cell death ± SD of three independent experiments (**D**). * *p* < 0.05, ** *p* < 0.01.

Both HSV-1 and Sendai virus strongly induce apoptosis and necroptosis [23]. We finally tested if A179L also regulates influenza virus-induced cell death in NL20 cells, a human bronchial epithelial cell line. As shown in Figure 6A, influenza virus infection cleaved PARP, caspase-8, and caspase-3 in a dose-dependent manner in the pcDNA3.1-transfected NL20 cells. A179L inhibited IAV-induced PARP, caspase-8, and caspase-3 cleavage (Figure 6B). Flow cytometric analysis revealed that pcDNA3.1 or A179L transfection alone did not significantly affect apoptosis, as the number of Annexin V-positive cells was almost equivalent to that in the untransfected control (Figure 6C). A179L transfection significantly decreased the number of apoptotic cells in IAV-infected NL20 cells, compared to those transfected with the pcDNA3.1 vector control (Figure 6C,D).

### 3.7. A179L Enhances Influenza Virus-Induced Necroptosis

**Figure 7:**
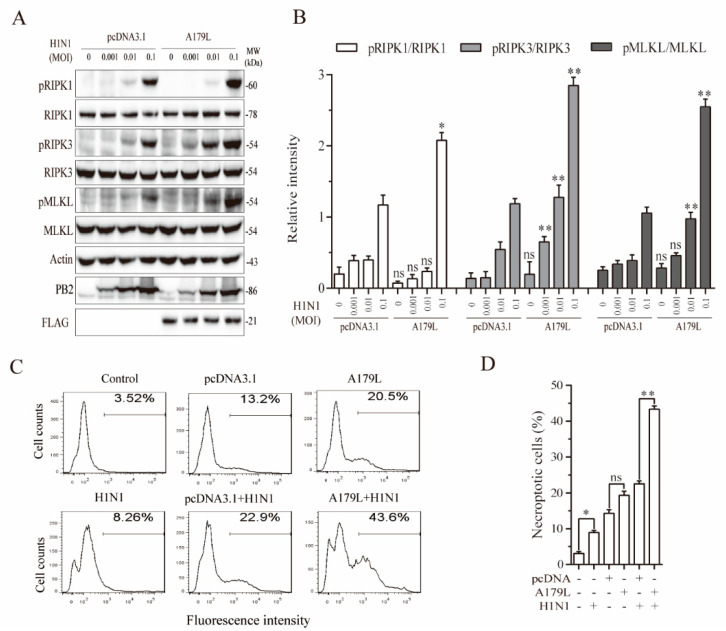
A179L enhanced IAV-induced necroptosis. (**A**) NL20 cells seeded in a 12-well plate were transiently transfected with pcDNA3.1 or the A179L expression vector. After incubation for 24 h, the cells were left uninfected or infected with the indicated dose of IAV and then incubated for 24 h. Cell lysates were prepared and analyzed for the levels of RIPK1^S166^, RIPK3, and MLKL and their corresponding total proteins. A179L expression was analyzed by Western blot with an anti-FLAG antibody. IAV infection was monitored by Western blot with an anti-PB2 antibody. The density of the bands was analyzed by using NIH Image-J software and normalized by the arbitrary units of their corresponding total protein or β-actin. Data are the mean ± SD of three experiments (**B**). * p < 0.05, ** p <0.01, compared to the pcDNA3.1 control; ns, not significant. (**C**) NL20 cells seeded in a 6-well plate were left untransfected or were transiently transfected with pcDNA3.1 or the indicated amount of the A179L expression vector (4 μg each). After incubation for 24 h, the cells were left uninfected or infected with IAV (1 MOI) for 24 h. Single-cell suspensions were stained with Sytox green and analyzed for necroptosis by flow cytometry (**C**). The results represent the mean percent of cell death ± SD of three independent experiments (**D**). * *p* < 0.05, ** *p* < 0.01.

Recent studies have shown that ZBP1 plays an important role in mediating influenza virus-induced necroptosis [17,40,41]. We tested whether A179L was also able to promote ZBP1-dependent necroptosis in NL20 cells. As shown in Figure 7A, IAV infection dose-dependently increased the levels of RIPK1, RIPK3, and MLKL phosphorylation in pcDNA3.1-transfected L929 cells. A179L increased the levels of IAV-induced RIPK1, RIPK3, and MLKL phosphorylation, compared to the pcDNA3.1-transfected corresponding controls (Figure 7B). Unlike HSV-1 and Sendai virus, IAV did not induce the cleavage of RIPK1 and RIPK3 proteins in NL20 cells and did not decrease the levels of A179L protein (Figure 7A). Of note, RIPK1 phosphorylation was detected as a truncated 60-kDa protein. Interestingly, several prior studies have shown the presence of this truncated 60-kDa RIPK1 in RIPK1-transfected 293T cells or in murine bone marrow-derived macrophages in various settings [42,43,44]. Flow cytometric analysis of Sytox green-stained cells revealed that pcDNA3.1 and A179L transfection alone weakly and moderately increased the number of necroptotic NL20 cells, respectively (Figure 7C,D). IAV infection alone weakly increased the number of Sytox green-positive cells. However, A179L transfection dramatically increased the number of necroptotic cells following IAV infection, compared to the pcDNA3.1 vector control infected with IAV (Figure 7C,D).

## 4. Discussion

Programmed cell death is considered the first-line defense of host cells to halt the growth of invading microbes [33]. The ASFV genome encodes many nonstructural protein genes that regulate immune responses and virulence [6]. A179L is a Bcl-2-like protein that binds the BH3-containing proteins such as Bid, Bim, Bak, and Bax, and is localized at the mitochondria and endoplasmic reticulum [28,29]. Several prior studies have shown that A179L is a potent inhibitor of apoptosis. It inhibits apoptosis in Vero cells transfected with the *tBid* gene [28], in K562 cells treated with cycloheximide plus actinomycin D [27], and in Sf9 insect cells infected with baculovirus [26]. Our present study focuses on the ability of A179L to regulate virus-induced apoptosis and necroptosis in mammalian cells. We provide evidence that A179L inhibited apoptosis but enhanced necroptosis induced by both DNA and RNA viruses and by TSZ. Our study unveils a previously unrecognized role of A179L in regulating cell death.

It is well established that inhibition of apoptosis facilitates virus replication in the early stage of virus infection [24,28]. However, cell death may increase virus release and benefit virus spread in the late stage of virus infection [14,18]. Necroptosis, a cellular process characterized by swelling and cell membrane rupture, promotes virus release [33]. In contrast, apoptosis is a cellular process characterized by cell membrane blebbing and nuclear shrinking [14,18]. The exposure of phosphatidylserine in the inner cell membrane of apoptotic cells to its receptor on macrophages facilitates phagocytosis, a process that kills viruses by engulfing whole infected cells [14,18]. Several prior studies have shown that A179L is a potent inhibitor of apoptosis [24]. Our present study demonstrates that A179L enhanced necroptosis induced by TNF-α, HSV-1 and two RNA viruses: Sendai virus and IAV. These observations suggest that A179L may enable ASFV replication by blocking apoptosis. Since A179L was able to enhance TNF-α-mediated necroptosis (Figure 1G), we speculate that A179L may facilitate virus release and spread at the late stage of the virus life cycle, if A179L can indeed promote necroptosis in a virus infection setting.

A179L is expressed as an 18-kDa protein at both the early and late post-infection times in macrophages [27]. A179L, which is localized at the mitochondria or endoplasmic reticulum, suppresses apoptotic cell death in different cellular systems. For example, A179L inhibits apoptosis induced by the double-stranded RNA-activated protein kinase (p68) in HeLa and BSC-40 cells [26]. It also suppresses apoptosis induced by protein synthesis inhibition in the human myeloid leukemia cell line K562 [27] and insect cells infected with a recombinant baculovirus, in a cell-anchorage-dependent manner [26]. Whether A179L inhibits virus-induced apoptosis in mammalian cells has not been reported. Our present study demonstrates that A179L decreased the levels of caspase 8 and caspase cleavage and the number of Annexin V-positive cells in IPEC-DQ infected with HSV-1 or Sendai virus, and in NL20 cells infected with IAV. These observations collectively suggest that A179L inhibits virus-induced apoptosis. Although the mechanisms by which these viruses induce apoptosis are complex, because it appears that they all activate caspase-8, we speculate that A179L may inhibit apoptosis by inhibiting caspase activation.

A179L is well conserved among different ASFV isolates and shares 94-99% amino acid homology across 179 amino acids encoded by the entire protein [6,24]. The central region of A179L contains a motif that shares great identity with the Bcl-2 homologous 3 (BH3) domain [29]. A179L is anchored to the mitochondrial outer membrane via a membrane anchoring sequence [30] and can bind to several BH-3 only proteins, including the activated truncated forms of the Bid protein, Bim, Puma, Bak, and Bax [29]. A179L binds these mitochondrial pro-apoptotic proteins and increases mitochondrial membrane permeability, thus releasing cytochrome C and inducing caspase 9 activation [24,29]. Our present study shows that A179L inhibited caspase 8 cleavage and its activity. Since caspase 8 is not activated by the mitochondrial pathway but rather by extrinsic stimuli such as TNF-α, Fas ligands, and Toll-like receptors [33], A179L likely suppresses caspase-8 activation independent of its activity on the BH3-containing pro-apoptotic proteins. How A179L inhibits caspase cleavage and activation remains unknown. Since ASFV induces caspase cleavage and activation in the late stage of virus replication, it is unlikely that A179L enhances necroptosis in infected cells by inhibiting caspase cleavage. Furthermore, A179L did not block the cleavage of RIPK1 and RIPK3 in HSV-1- and Sendai virus-infected cells (Figure 3A and Figure 5A). How A179L switches apoptosis to necroptosis remains to be investigated.

Several questions remain unanswered. First, whether A179L-deleted ASFV affects cell death in porcine macrophages remains to be investigated. 3D4 cells, a porcine macrophage cell line, do not express RIPK3 and cannot be used to investigate the effect of A179L on cell death (data not shown). Primary porcine macrophages could be used to investigate the role of A179L in cell death. Our ongoing investigation showed that ASFV infection increased MLKL phosphorylation in porcine alveolar macrophages. This preliminary evidence suggests that ASFV is capable of inducing necroptosis in relevant cell types. Second, the ability of A179L to enhance TNF-α and virus-induced necroptosis has not been verified in a virus infection setting. A179L by itself has been shown to block autophagosome formation [30,31]. However, A179L-deficient ASFV can still inhibit starvation-induced autophagy by mTOR activation, suggesting that A179L is dispensable for ASFV-mediated suppression of autophagy [32]. Similarly, other ASFV genes may overshadow or counteract the effect of A179L on necroptosis in a virus infection setting. It remains uncertain whether A179L plays a role in modulating cell death in ASFV-infected cells. Third, the mechanisms of A179L-enhanced necroptosis were not investigated. Nevertheless, our present study provides evidence that A179L inhibits virus-induced apoptosis but enhances necroptosis induced by TNF-α or TSZ and by both DNA and RNA viruses. These findings warrant further investigation of the role of A179L on cell death in an ASFV-based setting and preferentially in porcine pulmonary alveolar macrophages.

## Data Availability

The data presented in this study are available on request from the corresponding author. The data are not publicly available due to ethical issues.

## Supplementary Materials

The following are available online at https://www.mdpi.com/article/10.3390/v13122490/s1, File S1: Original Western blot images.
