# Supplementary figures and images for: The A179L Gene of African Swine Fever Virus Suppresses Virus-Induced Apoptosis but Enhances Necroptosis
Source: Viruses. 2021 Dec 13;13(12):2490. doi: 10.3390/v13122490 (PMC8708531; doi:10.3390/v13122490)

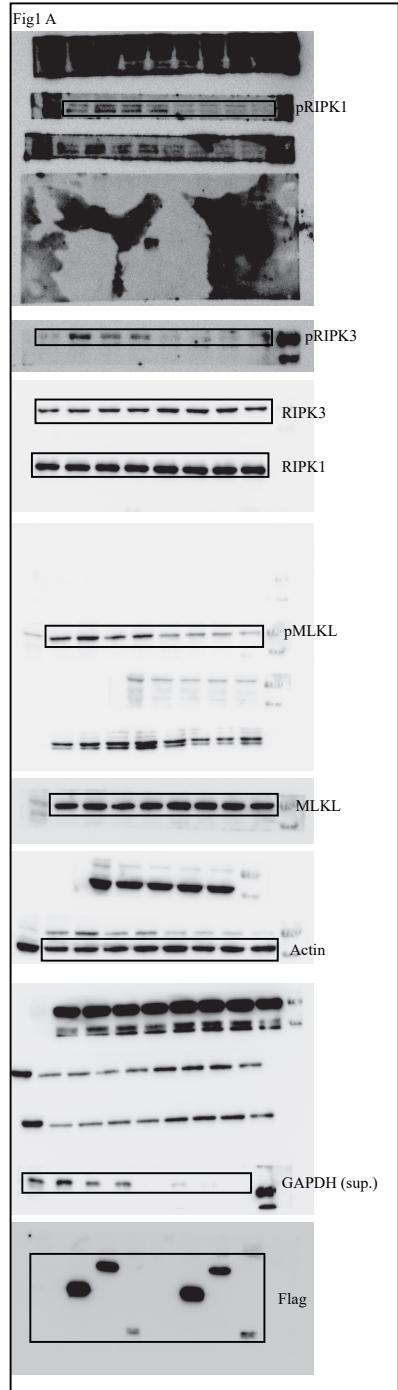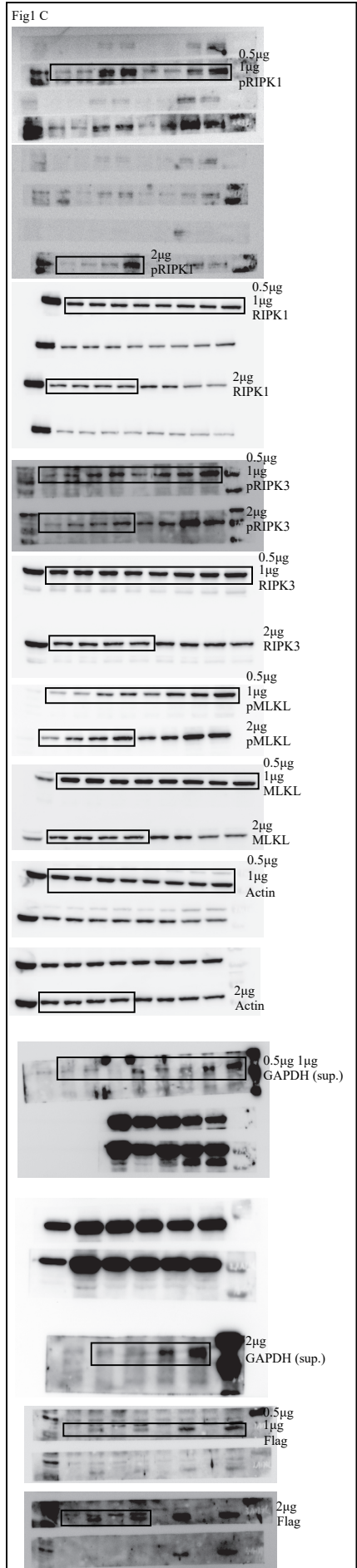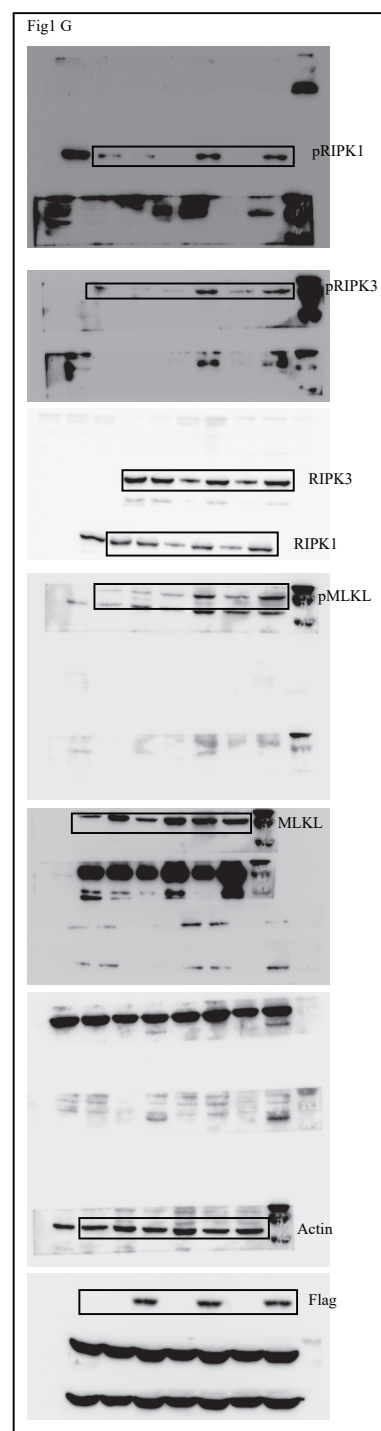

Fig2 A

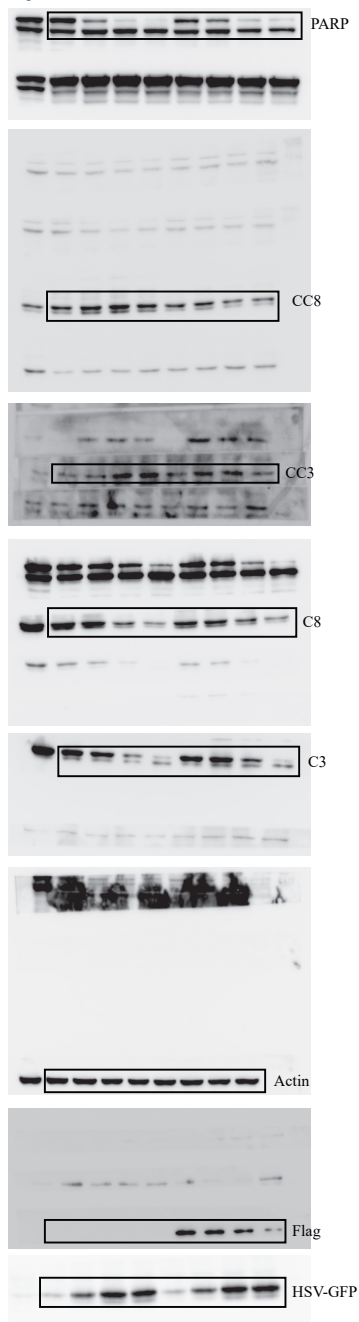

Fig3 A

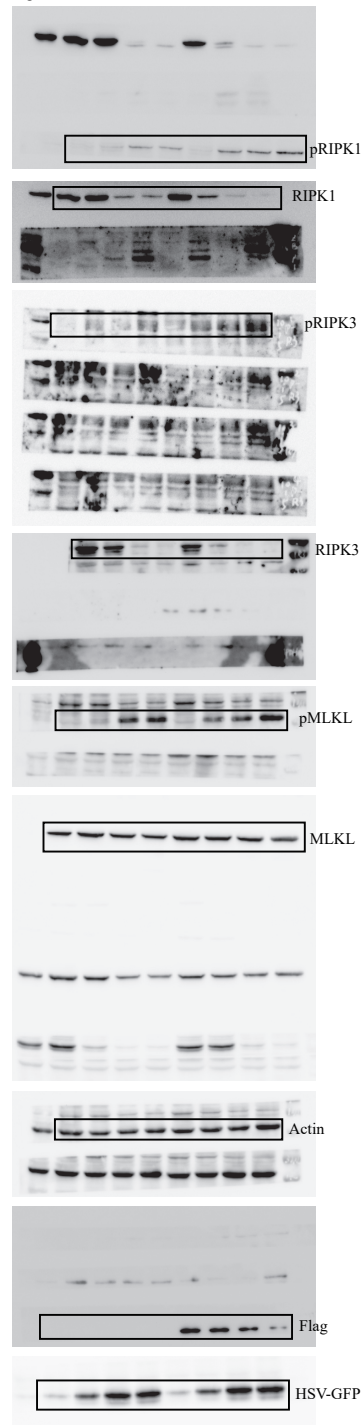

Fig4 A

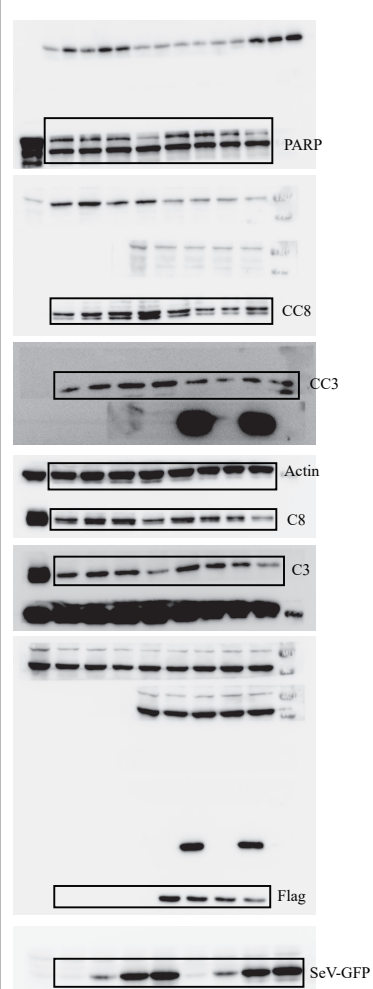

Fig5 A

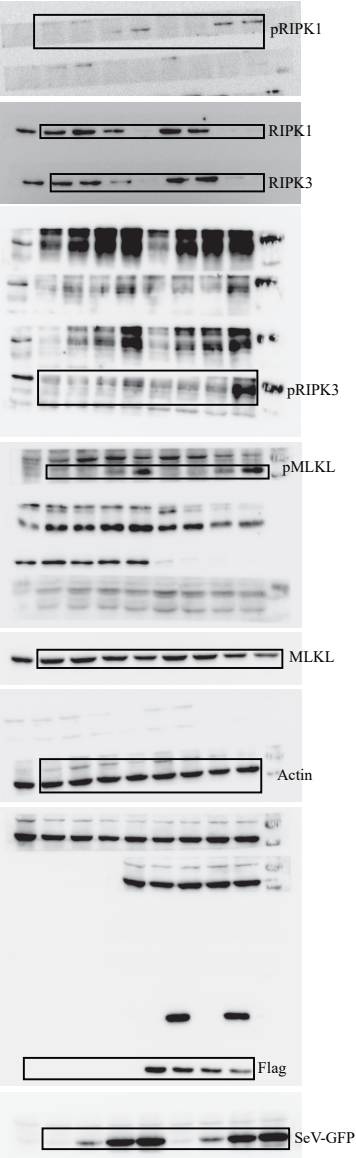

Fig6 A

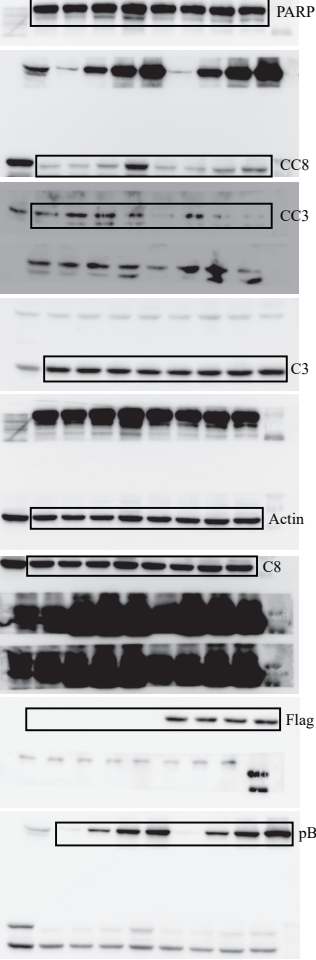

Fig7 A

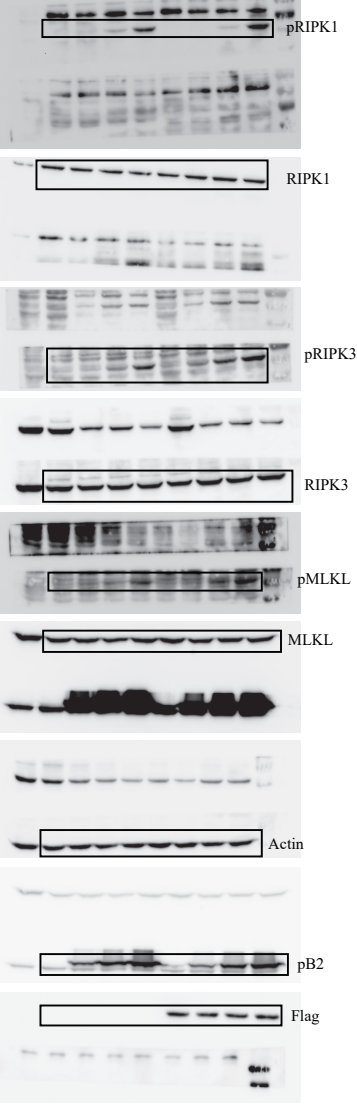

Supplement: Supplementary file 1 [file viruses-13-02490-s001.zip › viruses-1470683-supplementary.pdf]
